# Composition and dynamics of intestinal fungi during the postnatal 2 months of very low birth weight infants

**DOI:** 10.1007/s00431-023-05257-w

**Published:** 2023-10-31

**Authors:** Ting Wang, Yanbo Lu, Junhua Wu, Beirong Yu

**Affiliations:** 1 Health Science Center, Ningbo University, Ningbo, Zhejiang China; 2 Pediatrics, Ningbo Women and Children’s Hospital, Ningbo, Zhejiang China

**Keywords:** Very low birth weight infants, Intestinal, Fungi

## Abstract

It has been found that intestinal fungi play a role in the composition of the intestinal microecology and in the formation and development of the immunity during childhood. We investigated the gut fungi composition of preterm infants to analysis composition and dynamics of intestinal fungi during the postnatal 2 months of very low birth weight infants. We collected feces from 34 very low birth weight infants (VLBWI) and 28 preterm infants with birth weight >1500 g. We extracted total fungal DNA from feces and analyzed the composition of gut fungus through ITS sequencing. The fungal detectable rate in the experimental group peaked on day 3 (85.19%), then gradually decreased and started to show an increasing trend again by day 28. There were significant differences in the alpha diversity of intestinal fungus between VLBWI and controls, and the VLBWI had its own characteristics at different time points in richness and diversity. A total of 10 phylums and 342 genera were identified in all VLBWI samples. The dominant fungal phylum of the VLBWI group is Ascomycota (50.3%)and Basidiomycota (48.8%). The functional metabolic activity of the experimental group was lower than that of the control group.

*Conclusion*: The composition and abundance of VLBWI intestinal fungal showed several alterations during the first 2 months of life. The prediction of gut microbiota function suggests that intestinal metabolic function may be altered in VLBWI.

**Table:** 

**What is Known:** *• A limited number of studies has been found that symbiont fungi may be able to calibrate host immunological responses, promote development of peripheral lymphoid organs, promote T cell responses, and even may be associated with the development of certain diseases, such as inflammatory bowel disease (IBD), NEC, and allergic diseases. However, previous studies on intestinal microecology have mainly focused on adults while neglecting the role of fungi in the gut of children due to the much lower abundance of intestinal fungi than bacteria, limitations of techniques for detecting fungi, the difficulty of obtaining samples, and the absence of largescale reference databases.*	
**What is New:** *• In recent years, the discovery and development of fungal detection technologies such as 18s rDNA sequencing technology, Internal Transcribed Spacer(ITS), and DNA fingerprinting technology have further broadened the perspective on the impact of intestinal fungal exposure in early life.*

**What is Known:**

*• A limited number of studies has been found that symbiont fungi may be able to calibrate host immunological responses, promote development of peripheral lymphoid organs, promote T cell responses, and even may be associated with the development of certain diseases, such as inflammatory bowel disease (IBD), NEC, and allergic diseases. However, previous studies on intestinal microecology have mainly focused on adults while neglecting the role of fungi in the gut of children due to the much lower abundance of intestinal fungi than bacteria, limitations of techniques for detecting fungi, the difficulty of obtaining samples, and the absence of largescale reference databases.*

**What is New:**

*• In recent years, the discovery and development of fungal detection technologies such as 18s rDNA sequencing technology, Internal Transcribed Spacer(ITS), and DNA fingerprinting technology have further broadened the perspective on the impact of intestinal fungal exposure in early life.*

## Introduction

Human beings have a rich and unique microecology in the channels that connect them to the outside world, such as the skin, digestive tract, respiratory tract, oral cavity, and genitourinary tract [1]. Among them, the intestinal microbiota is the most representative microbial community [1]. Previous studies [2, 3] on intestinal microecology have mainly focused on intestinal bacteria or selected adults as subjects while neglecting the role of fungi in the gut of children due to the much lower abundance of intestinal fungi than bacteria, limitations of techniques for detecting fungi, the difficulty of obtaining samples, and the absence of large-scale reference databases. In particular, people still know little about the dynamic influence of fungi on the formation of intestinal microecological populations in neonates.

VLBWI, defined as newborns with birth weight <1500 g, are a special category of newborns characterized by low immunity and susceptibility to infectious diseases such as late-onset sepsis (LOS) and necrotizing enterocolitis (NEC) [4]. It has been reported that the development of complications such as feeding intolerance (FI), NEC, and LOS in VLBWI may be related to their unique gut microflora composition [5, 6].

A limited number of studies has been found that symbiont fungi may be able to calibrate host immunological responses [7, 8], promote development of peripheral lymphoid organs [9], promote T cell responses [10], and even may be associated with the development of certain diseases, such as inflammatory bowel disease (IBD), NEC, and allergic diseases [11–15].

There still are few studies on the intestinal fungi of very low birth weight infants. Based on the above-mentioned, first of all, we collected stool samples from 62 preterm infants at different time points, including 34 VLBWI and 28 preterm infants with birth weight >1500 g, then analyzed their intestinal fungi by ITS sequencing. At last, we explored the characteristics of intestinal fungi in VLBWI.

## Method

### Trial participants

After obtaining informed consent from parents, preterm infants who were admitted to the NICU directly after birth with a birth weight of less than 1500 g were eligible to participate in the experimental group, and preterm infants who had a birth weight of greater than 1500 g were eligible to participate in the control group. Exclusion criteria were preterm infants with severe asphyxia at birth or had congenital malformations, genetic metabolic disorders, or the presence of shock or multi-organ failure. The trial was in line with the Declaration of Helsinki and approved by the Ethics Committee of Ningbo Women’s and Children’s Hospital, Zhejiang Province, China.

### Sampling

Medical personnel collected 2 g of stool samples on day 1, day 3, day 7, day 14, day 21, day 28, and day 60 (experimental group only) after the admission of study subjects who met the criteria with a special specimen box for stool cleaning, and stored them immediately in a −80 °C refrigerator. Collection was stopped for study subjects who were discharged or died in the middle of the study.

### DNA extraction and sequencing of the ITS genes

Microbic DNA was extracted by a QIAamp DNA Stool Mini Kit (Qiagen) according to the manufacturer’s instructions. Then, DNA was quantified by Nanodrop, and the quality of DNA extraction was assessed by 1.2% agarose gel electrophoresis. The fungal-specific gene fragment sequence was amplified by the universal fungal primers. The purification of polymerase chain reaction PCR products was performed using Vazyme VAHTSTM DNA Clean Beads, and Illumina bridge PCR-compatible primers were introduced. The amplified products were detected by Quant-iT PicoGreen dsDNA Assay Kit fluorescent reagent and Microplate reader (BioTek, FLx800) quantification instrument. Sequencing libraries were prepared using Illumina’s TruSeq Nano DNA LT Library Prep Kit, and finally, the resulting samples were subjected to ITS high-throughput sequencing.

The raw data obtained from the Illumina platform turned into effective Amplicon Sequence Variants (ASVs) after removing the primer sequence, trimming, merging, and filtering. The alpha diversity refers to the fungal diversity within each sample, and it was calculated by using Simpson’s reciprocal index, which describes how many ASVs prevail in each sample. The beta diversity expresses the difference between the samples in terms of the number and abundance of ASVs within an age group, and it was calculated with the Bray-Curtis dissimilarity index, predicting the metabolic function of a sample’s fungus, identifying differential pathways, and obtaining the species composition of specific pathways. Functional prediction of ITS gene sequences in the MetaCyc database using PICRUSt2 was followed by functional unit PCoA analysis, i.e., using Bray-Curtis distance matrix combined with principal coordinates analysis to expand sample functional differences in low dimensions. After obtaining the abundance data of metabolic pathways, we used Student’s *t*-test to try to identify metabolic pathways with significant differences between groups.

### Statistical analyses

Student’s *t*-test and analysis of variance were performed for the measurement data, and chi-square test analysis was performed for the count data by SPSS 23.0. Graphpad Prism v.8.0.2. was used for plotting.

## Result

### Participants’ characteristics

Sixty-two preterm infants were divided into two groups according to birth weight, including 34 VLBWI and 28 preterm infants with birth weight > 1500 g. There were no differences between the two groups in terms of sex (*t* = 0.01, *P* = 0.921) and delivery mode (*t* = 0.01, *P* = 0.921). However, there were significant differences in terms of gestational age, preterm rupture of membranes, amniotic fluid contamination, mechanical ventilation, and central vein, and the positive results are shown in Table 1.

**Table 1:** Participants’ characteristics

		Experimental group	Control group	t/c2	*P*
Premature rupture of membrane	Yes	9	1	4.38^a^	0.036
	No	25	27		
Apnea	Yes	3	4	36.927^a^	< 0.001
	No	31	24		
Amniotic fluid contamination	Yes	2	2	46.901^a^	< 0.001
	No	32	26		
Mechanical ventilation	Yes	26	6	18.627^a^	< 0.001
	No	8	22		
Central venous catheter	Yes	34	9	33.266^a^	< 0.001
	No	0	19		
Gestational age (weeks)		29.9 ± 2.22	33 ± 1.32	− 6.833^b^	< 0.001
Birth weight(g)		1239.41 ± 172.64	1896.43 ± 289.43	− 11.07^b^	< 0.001

^a^*χ*^2^ calculations

^b^Student’s *t*‐test

### Data quality control and ASV cluster

**Fig. 1:**
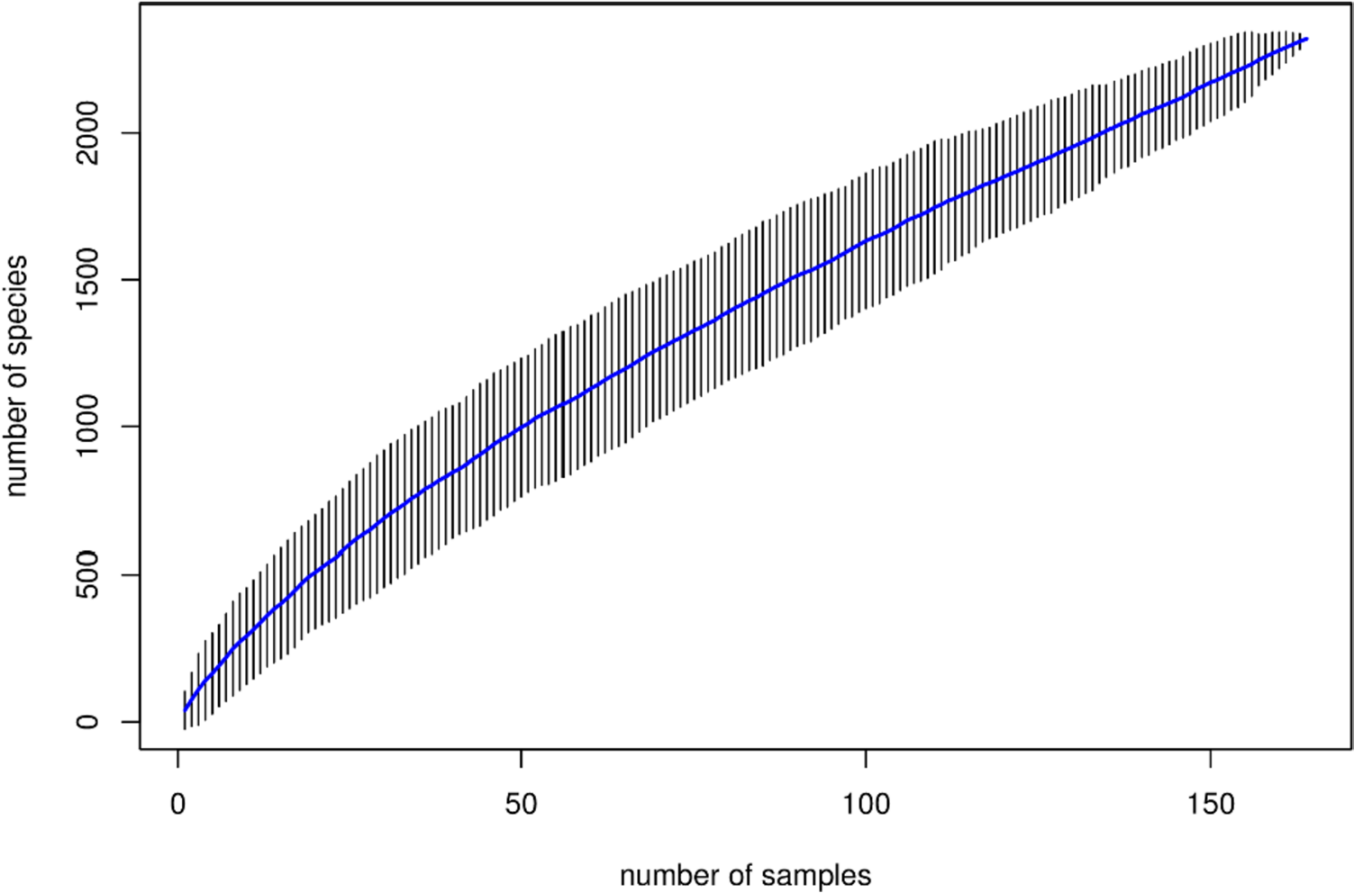
Specaccum species accumulation curves: sample size is on the abscissa, the number of observed species (ASVs) is on the ordinate, and the shading reflects the confidence intervals of the curves. The results reflect the rate of addition of new species observed when the sample size is continuously expanded over the course of sampling the population of the sample

A total of 15,222,063 original sequences were obtained. After removing low-quality sequences and denoising, 87.45% (13,311,092/15,222,063) of valid sequences were retained. There were 9,431,961 sequences in the experimental group with 84,213.93 sequences on average for each sample, and 3,938,448 sequences in the control group with 75,739.38 sequences on average, and the number of denoised sequences in the two groups was significantly different by Student’s *t*-test (*t* = 2.08, *P* = 0.039). Specaccum species accumulation curves (Fig. 1) were plotted for the total number of ASVs whose end of the curve was flattened out, indicating that the sample size was sufficient to reflect the species composition of the community.

**Fig. 2:**
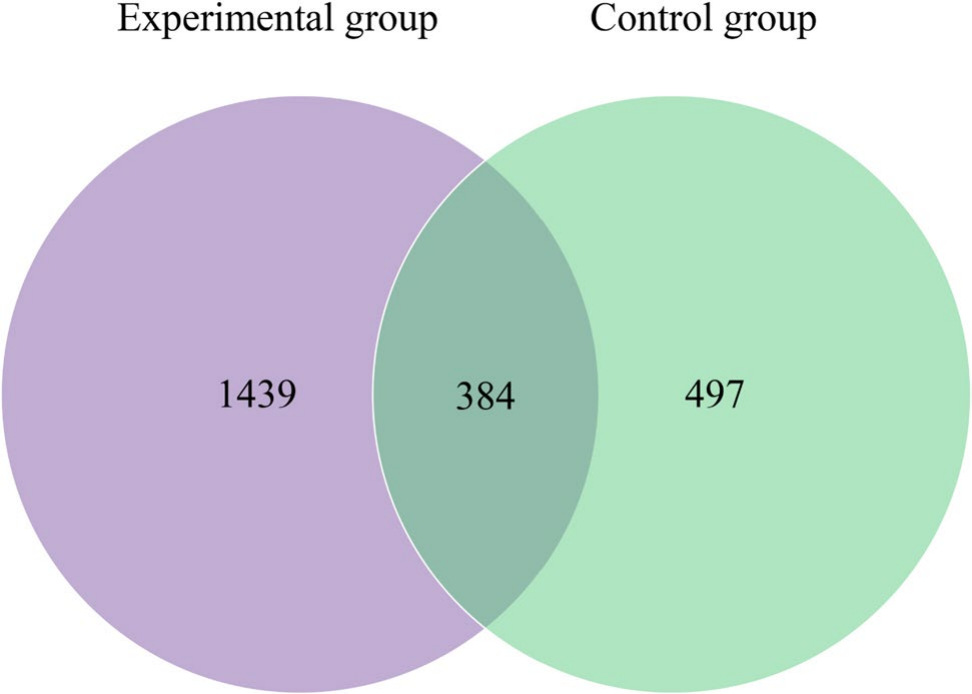
Venn diagram of species differences between the two groups

All valid sequences were clustered into ASVs at 100% similarity, and the ASV abundance was counted for each sample, a total of 2704 ASVs were detected in the two groups of samples. The unique ASVs accounted for 62.03% (1439/2320) of all ASVs in the experimental group and 21.42% (497/2320) in the control group. 16.55% (384/2320) ASVs were common to the two groups (Fig. 2). This result suggested that the two groups have their own characteristics in the ASVs.

### Detectable rate

**Fig. 3:**
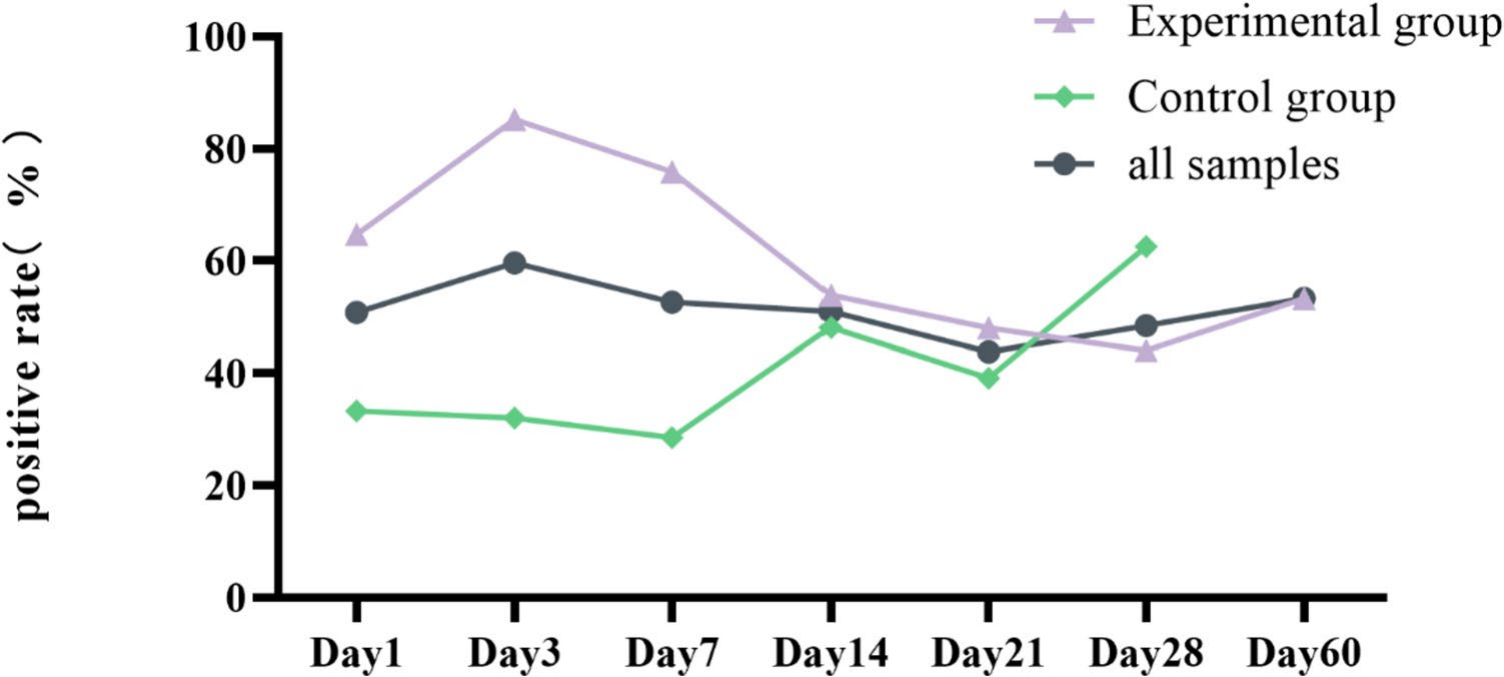
Folding line graph of fungal detectable rate in each group

Successful annotation to fungal taxa was obtained from 164 samples from 319 samples of participants. The positive rate of all samples is 51.41% (Table 2). The fungal detectable rate was 61.88% (112/181) for the experimental group, which was significantly higher than 37.68% (52/138) for the control group (*t* = 13.599, *P* < 0.001) (Table 3). Among the time points, there was no statistically significant difference in the detection rates between the experimental group and the control group at day 14 (*t* = 1.175, *P* = 0.278), day 21 (*t* = 0.383, *P* = 0.536), and day 28 (*t* = 0.830, *P* = 0.362), whereas the detection rates of fungi in the experimental group at days 1, 3, and 7 were significantly higher than those in the control group and the specific positive results are shown in Table 3. The fungal detectable rate in the experimental group peaked on day 3 (85.19%), then gradually decreased and started to show an increasing trend again by day 28, while the detection rate of fungi in the control group was relatively stable within the first week of life and then showed an overall upward trend. However, the overall detection rate maintained a stable trend over time (Fig. 3).

**Table 2:** Fungal detectable rate of all samples in different time

	Total sample	Detectable sample	Detectable rate (%)
Day 1	61	31	50.820
Day 3	52	31	59.620
Day 7	57	30	52.630
Day 14	53	27	50.940
Day 21	48	21	43.750
Day 28	33	16	48.480
Day 60	15	8	53.330
Total	319	164	51.410

**Table 3:** The counts of samples with detected and non-detected fungal DNA for two groups

	Experimental group			Control group			*t*	*P*
	Negative (*n*)	Positive (*n*)	Positive rate (%)	Negative (*n*)	Positive (*n*)	Positive rate (%)		
D1	12	22	64.710	18	9	33.330	5.926	0.015
D3	4	23	85.190	17	8	32	15.251	< 0.001
D7	7	22	75.860	20	8	28.570	12.779	< 0.001
Summary	69	112	61.880	76	52	37.680	13.599	< 0.001

### Analysis of species composition

#### Alpha diversity

**Fig. 4:**
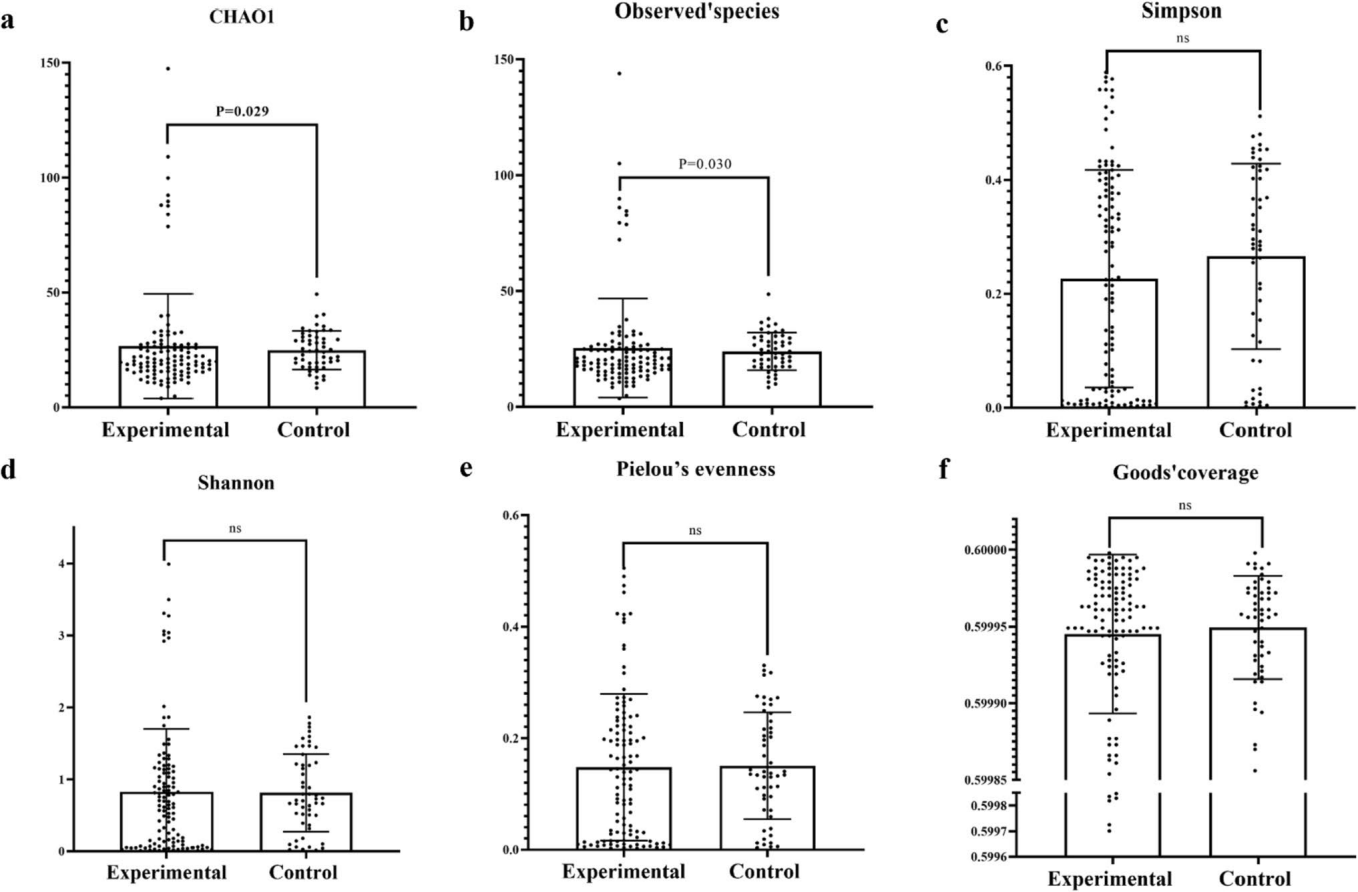
**a**–**f** The box plots comparing the abundance between the two groups. **a** Chao1; **b** Observed species; **c** Simpson; **d** Shannon; **e** Pielou’s evenness; **f** Goods’ coverage

**Fig. 5:**
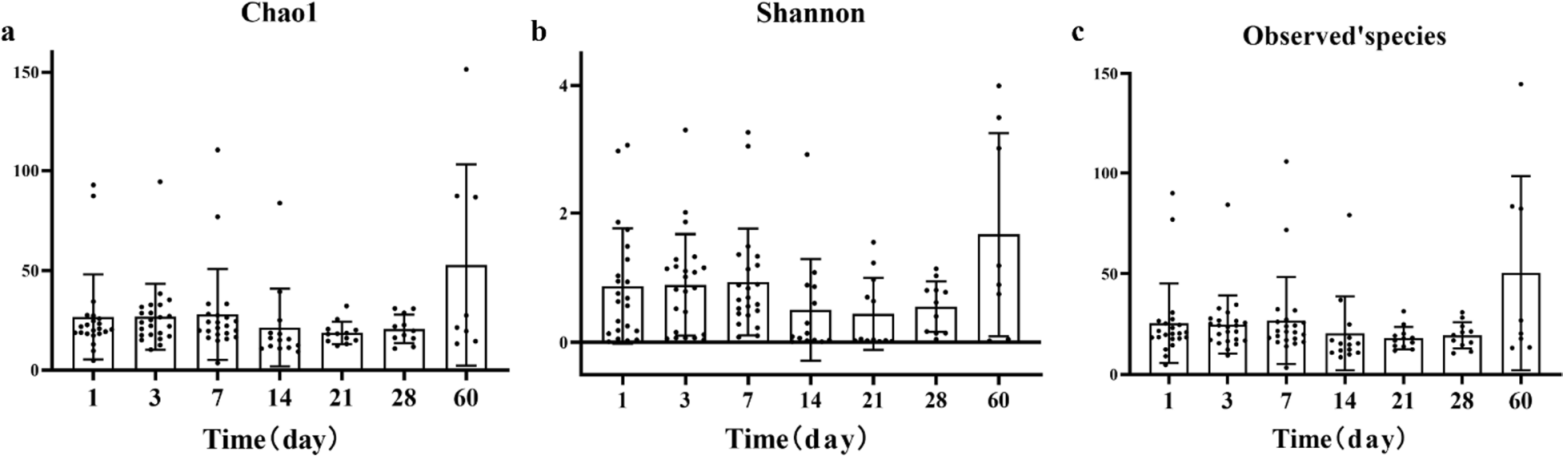
**a**–**c** The box plots comparing the abundance between different time points in VLBWI. **a** Chao1; **b** Simpson; **c** Observed species

The Alpha diversity indices used in our study include Chao1, Simpson, Shannon, Pielou’s evenness, Observed species, and Goods’ coverage. Richness was characterized by Chao1 and Observed species; diversity was characterized by Shannon and Simpson; evenness was characterized by Pielou’s evenness; depth was characterized by Good’s coverage. By Student’s *t*-test of the diversity indices of the two groups, Chao1 and Observed species were significantly higher in the experimental group than in the control group (Table 4), suggesting that the species richness of the experimental group was significantly higher than that of the control group (Fig. 4a–f). Analyzing the alpha diversity results by ANOVA in the VLBWI group found that there are significant differences in Chao1, Shannon, and Observed species at different time points (Table 5), suggesting that the VLBWI had its own characteristics at different time points in richness and diversity. Further comparisons revealed that there were no significant difference between days 1, 3, 7, 14, 21, and 28 within the VLBWI group, while the alpha diversity at day 60 was significantly higher than at other time points (Fig. 5a–c).

**Table 4:** The alpha diversity in two groups

	Experimental group	Control group	*P*
Chao1	26.580 ± 22.777	24.857 ± 8.418	0.029*
Simpson	0.226 ± 0.191	0.266 ± 0.163	0.177
Shannon	0.824 ± 0.877	0.813 ± 0.539	0.252
Pielou’s evenness	0.148 ± 0.132	0.151 ± 0.096	0.416
Observed species	25.363 ± 21.397	23.946 ± 8.124	0.030*
Goods’ coverage	0.600 ± 0.00005	0.600 ± 0.00003	0.645

*P < 0.05

**Table 5:** Distribution of alpha diversity at different points in time of VLBWI

	Chao1	Simpson	Shannon	Pielou’evenness	Observed’species	Goods’coverage
Day 1	26.924 ± 22.527	0.239 ± 0.197	0.872 ± 0.890	0.155 ± 0.136	25.45 ± 19.917	0.59994 ± 0.000063
Day 3	26.384 ± 17.439	0.262 ± 0.191	0.894 ± 0.786	0.162 ± 0.126	24.843 ± 14.926	0.59994 ± 0.000066
Day 7	28.088 ± 23.685	0.259 ± 0.171	0.935 ± 0.824	0.172 ± 0.119	26.818 ± 21.940	0.59994 ± 0.00005
Day 14	21.212 ± 19.813	0.141 ± 0.186	0.507 ± 0.790	0.096 ± 0.126	20.621 ± 18.797	0.59996 ± 0.00004
Day 21	18.654 ± 5.616	0.135 ± 0.175	0.444 ± 0.559	0.087 ± 0.107	18.1 ± 5.552	0.59996 ± 0.00002
Day 28	20.361 ± 7.125	0.179 ± 0.151	0.557 ± 0.391	0.112 ± 0.078	19.527 ± 6.470	0.59995 ± 0.00003
Day 60	52.848 ± 49.508	0.349 ± 0.240	1.676 ± 1.582	0.253 ± 0.207	50.463 ± 48.178	0.59991 ± 0.00006
*F*	2.399	1.944	2.372	2.094	2.501	1.142
*P*	0.033*	0.080	0.034*	0.060	0.027*	0.343

#### Beta diversity

**Fig. 6:**
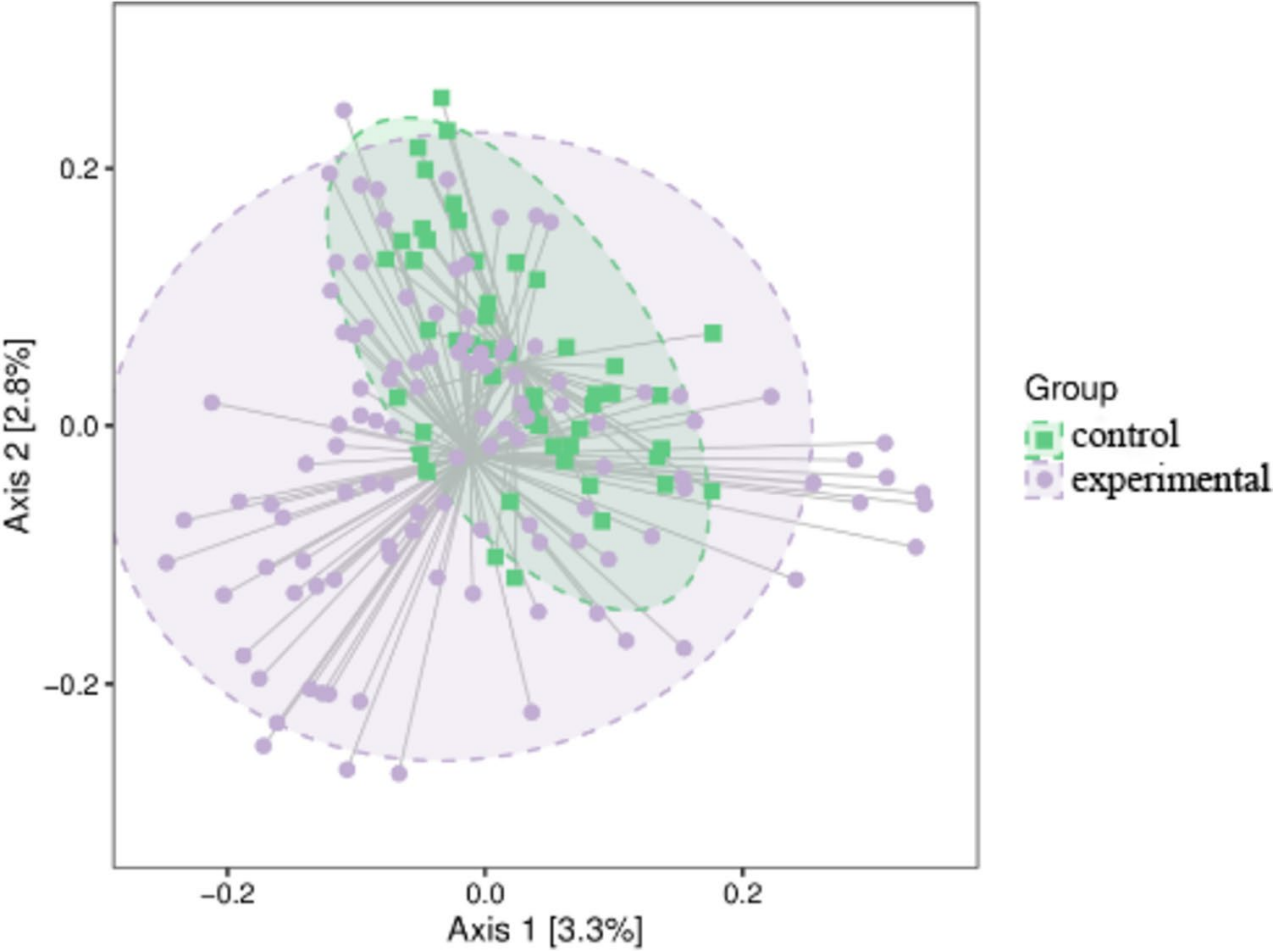
Two-dimensional sorting chart of samples for PCoA analysis. Each point in the figure represents a sample and points of different colors indicate different samples (groups). The closer the projection of two points on the axes, the more similar the community composition of these two samples in the corresponding dimension. The elliptical dashed circle refers to the 95% confidence ellipse (i.e., 95 out of 100 samples in this sample group will fall in it)

PcoA analysis (Fig. 6) was performed, suggesting a trend of clustering of intestinal fungi between the two groups. Significance assessment of two groups using PERMANOVA revealed a significant difference in the distribution of intestinal fungal composition between the two groups (*t* = 1.85256, *P* = 0.008).

### Overview of the gut microbiota composition and detective rate in VLBWI

**Fig. 7:**
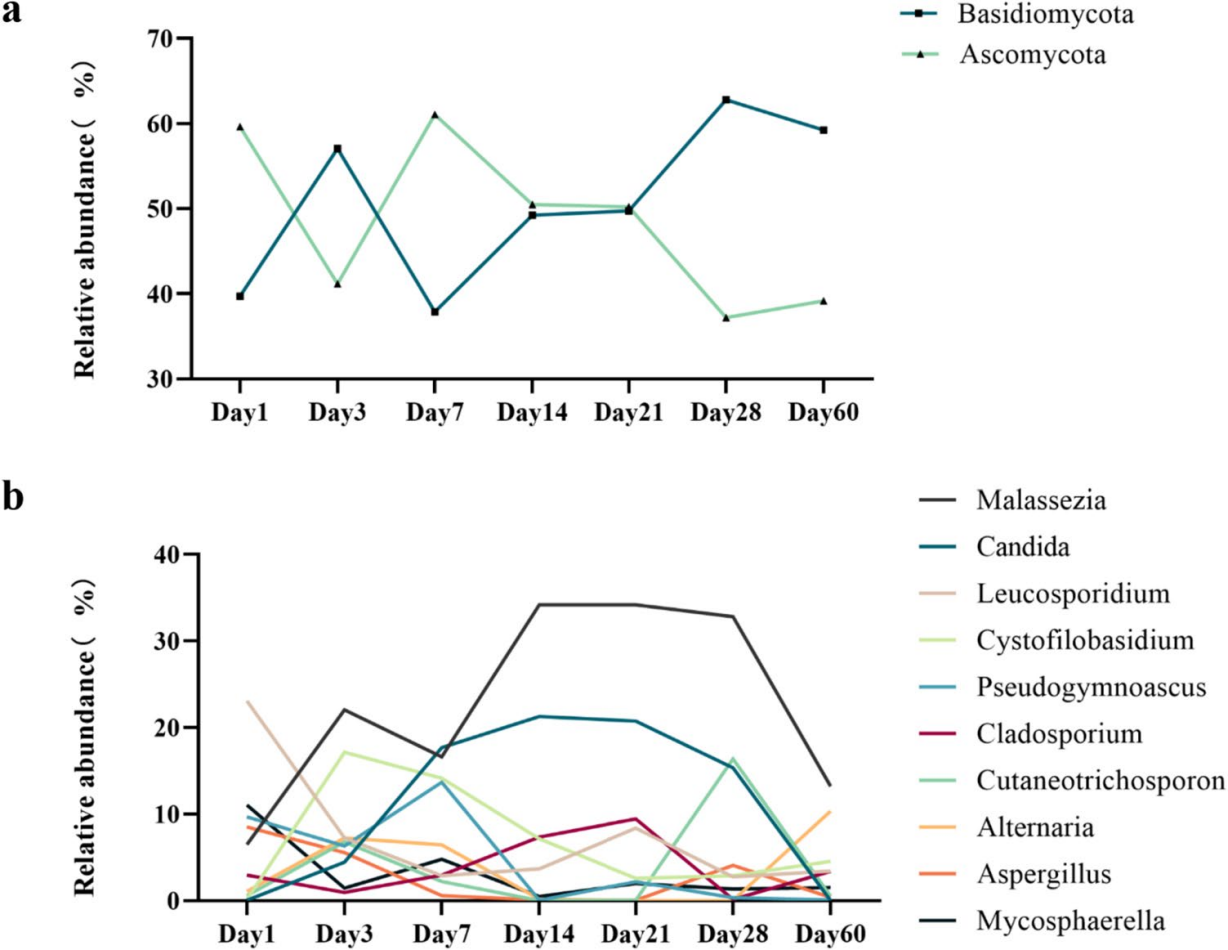
**a**, **b** Folding line graph of fungal abundance in VLBWI at different time points **a** in the level of phylum; **b** in the level of genus

A total of 10 phylums and 342 genera were identified in all VLBWI samples. The ten fungi that were detected were Ascomycota, Basidiomycota, unclassified_Fungi, unidentified, Mucoromycota, Mortierellomycota, Chytridiomycota, Basidiobolomycota, Glomeromycota, and Blastocladiomycota. The top two fungi in phylums, Ascomycota (50.3%), Basidiomycota (48.8%), comprised 99.1% of the fungal population abundance, who showed a zigzag pattern of change in the first 2 weeks. Ascomycota abundance was higher on days 1 and 7, lower on days 3 and 14, and then entered a more stable period, with an overall decreasing trend after day 21. However, the trend of Basidiomycota was just opposite to that of Ascomycota (Fig. 7a).

At the genus level, the top 50 genera in abundance accounted for 97.38% of the total fungal genera detected. The top ten dominant genera in abundance were *Malassezia* (21.2%), *Candida* (10.8%), *Leucosporidium* (8.45%), *Cystofilobasidium* (8.11%), *Pseudogymnoascus* (6.17%), *Mycosphaerella* (3.92%), *Alternaria* (3.74%), *Cutaneotrichosporon* (3.60%), *Cladosporium* (3.54%), and *Aspergillus* (3.37%) (Fig. 7b). Their combined abundance accounted for 72.9% of the total genera. The abundance of *Malassezia* and *Candida*, as the two dominant fungi, showed similar trends, with an overall increasing trend within 2 weeks after birth, then peaked in 2 weeks and a leveling off from 2 weeks to 28 days before starting to decline. A similar trend was observed for *Cladosporium* but differed in that it started to show an increasing trend again at day 60. The abundance of *Cystofilobasidium* and *Pseudogymnoascus* increased to a high level in the early stage, but after 1 week of birth, it had a decreasing trend over time. *Leucosporidium* were present at relatively high abundance in samples retained on day 1 after birth but showed an overall decreasing trend over time. At day 28, most fungal genera began to show a decreasing trend or reached a trough, while *Cutaneotrichosporon* reached a peak. *Alternaria*, *Aspergillus*, and *Mycosphaerella* maintained low abundance levels with small fluctuations throughout the observation period (2 months after birth).

### Fungal variance analysis

**Fig. 8:**
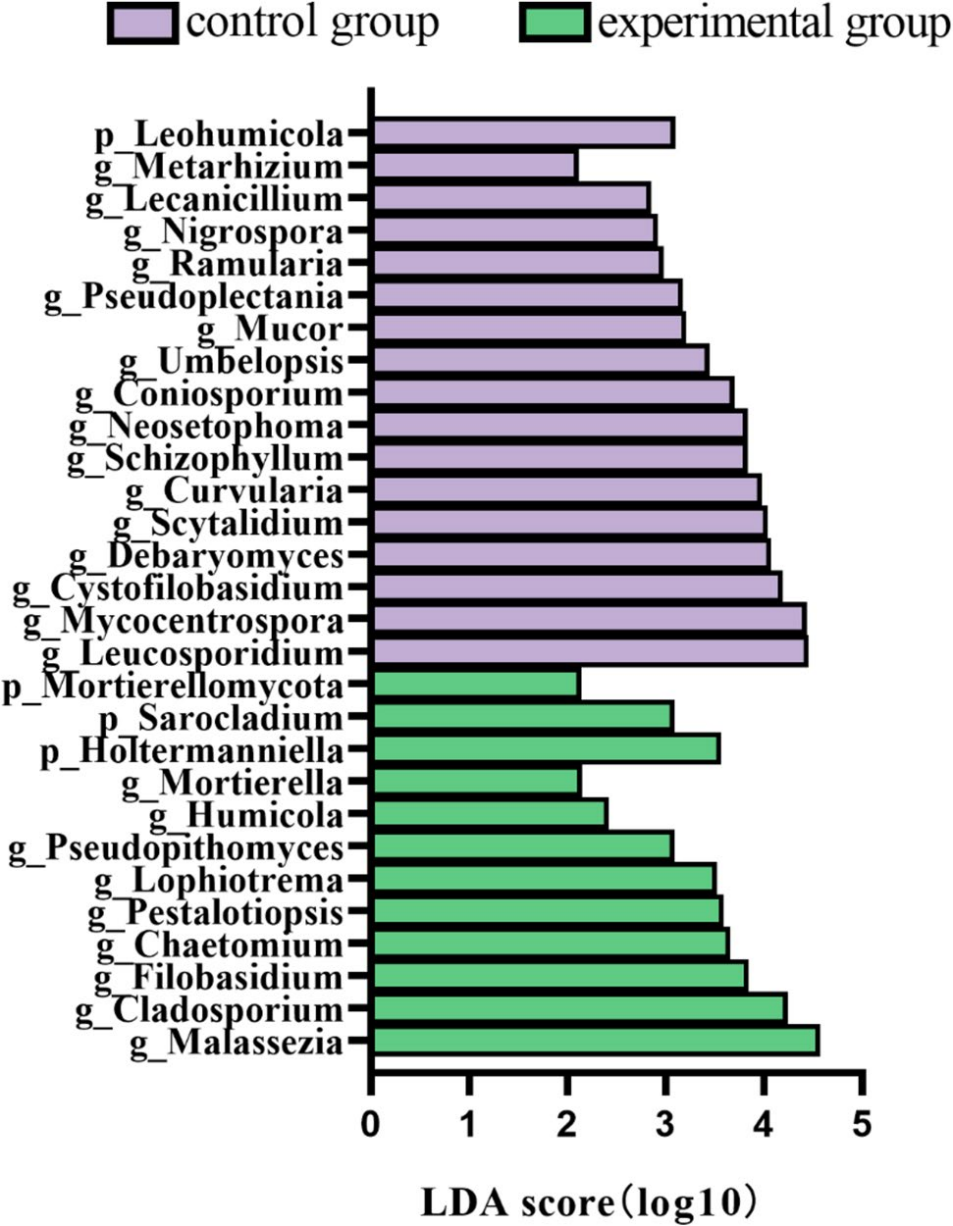
Histogram of LDA effect values for marker species. The vertical coordinates are the categorical units with significant differences between groups, and the horizontal coordinates visualize the logarithmic score of the LDA analysis for each categorical unit in a bar chart. The classification units are ranked according to the size of their scores, which describes their specificity in the sample grouping. Longer lengths indicate more significant differences in the classification units

Species difference analysis was performed on the phyla and genera between the two groups, and a total of 4 phyla and 25 genera with significant differences were detected. Ranking according to the significance of the differences from least to most. At the phylum level, the abundance of Mortierellomycota, Sarocladium, and Holtermanniella was significantly higher and Leohumicola was significantly lower in the experimental group. At the genus level, the abundance of *Mortierella*, *Humicola*, *Pseudopithomyces*, *Lophiotrema*, *Pestalotiopsis*, *Chaetomium*, *Filobasidium*, *Cladosporium*, and *Malassezia* was significantly higher in the experimental group, and the significantly higher abundance of *Umbelopsis*, *Coniosporium*, *Neosetophoma*, *Schizophyllum*, *Curvularia*, *Scytalidium*, *Debaryomyces*, *Cystofilobasidium*, *Mycocentrospora*, *Leucosporidium* was significantly lower (Fig. 8).

### Fungal community functions differ according to gestational age

**Fig. 9:**
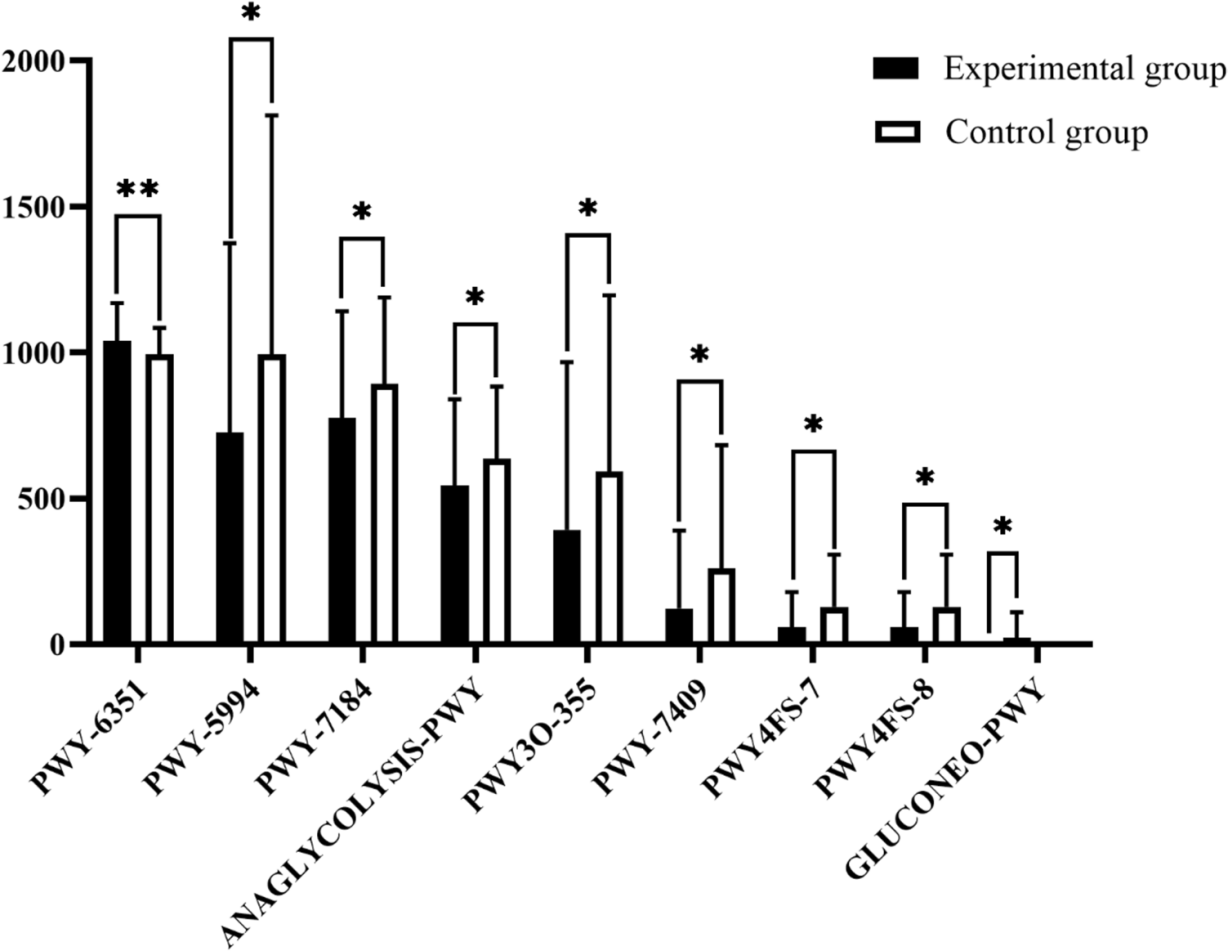
Predictive function analysis of KEGG pathways for the fecal fungus

The ITS feature sequences were aligned with the reference sequences to construct the evolutionary tree. Using the Castor algorithm, the nearest sequence species of the feature sequences were inferred based on the gene family copy number corresponding to the reference sequence in the evolutionary tree, and thus their gene family copy numbers were obtained. The gene family copy number of each sample is calculated by combining the abundance of each sample. Finally, the gene families were “mapped” to the MinPath database to infer the presence of metabolic pathways, and the abundance of metabolic pathways in each sample was obtained, and the MetaCyc database was used for metabolic pathway prediction. We compared the two sets of results and found that there were 9 differences between two groups in functional abundances, which were in glycolysis III (*P* = 0.042), gluconeogenesis I (*P* = 0.013), palmitate biosynthesis I (*P* = 0.024), D-myo-inositol (1,4,5)-trisphosphate biosynthesis (*P* = 0.01), pyrimidine deoxyribonucleotides de novo biosynthesis I (*P* = 0.043), phospholipid remodeling (*P* = 0.035), stearate biosynthesis III (*P* = 0.044), phosphatidylglycerol biosynthesis I (*P* = 0.013), and phosphatidylglycerol biosynthesis II (*P* = 0.013) (Table 6). We found that the expression of all function pathways was reduced in the experimental group compared to the control group, except for gluconeogenesis I, D-myo-inositol (1,4,5)-trisphosphate biosynthesis (Fig. 9). This result suggests that the functional metabolic activity of the experimental group was lower than that of the control group.

**Table 6:** Names of metabolic pathways and metabolites

Coding of pathways	Pathway metabolic function
ANAGLYCOLYSIS-PWY	Glycolysis III (from glucose)
GLUCONEO-PWY	Gluconeogenesis I
PWY-5994	Palmitate biosynthesis I (animals and fungi)
PWY-6351	D-myo-inositol (1,4,5)-trisphosphate biosynthesis
PWY-7184	Pyrimidine deoxyribonucleotides de novo biosynthesis I
PWY-7409	Phospholipid remodeling (phosphatidylethanolamine, yeast)
PWY3O-355	Stearate biosynthesis III (fungi)
PWY4FS-7	Phosphatidylglycerol biosynthesis I (plastidic)
PWY4FS-8	Phosphatidylglycerol biosynthesis II (non-plastidic)

## Discussion

Previous studies have mainly investigated the composition and role of intestinal bacterial communities, but little is known about fungal communities, especially about the long-term effects of fungi on the formation of early intestinal microecological populations [16]. However, intestinal fungi are a non-negligible part of the intestinal microbiota [17]. It has been found that colonization of neonates by intestinal fungi may not only be associated with the development of invasive fungal disease, but may also be involved in the establishment and maturation of the human immune system [12, 18–20], which may even be associated with the development of certain future diseases.

In this study, 34 VLBWI and 28 preterm infants with a birth weight of more than 1500 g who were hospitalized in Ningbo Women’s and Children’s Hospital from January 2021 to April 2022 were enlisted. The two groups differed in terms of gestational age, birth weight, preterm rupture of membranes, asphyxia, amniotic fluid contamination, mechanical ventilation, and central veins. Disease severity tends to be negatively correlated with gestational age, so gestational age can be associated with many covariates and potential factors of disease associated with preterm birth [21], which lead to these differences in participant’s characteristics between the two groups. By collecting stool samples from these participants at 7 time points in the experimental group and 6 time points in the control group for sequencing, trends in their intestinal fungal composition and dynamics were described. The result of quality control indicated that the sample size of this study was sufficient for data analysis, and the sequencing depth met the requirements.

Willis et al. [2] were able to detect Microeukaryotes (mainly of fungal origin) in the first meconium sample after birth, suggesting that the presence of intestinal fungi may retrace to the fetal period. In the present study, the fungal detectable rate was 50.82% on the first day for all stool samples and up to 64.71% for the VLBWI, which corroborate the above findings.

Experimental results shown that the fungal detectable rate was significantly higher in the experimental group (*χ*^2^ = 13.59, *P* =  < 0.001). Microbial infestation in the uterine cavity may cause preterm labor [22], such as intrauterine inflammation. And as a fungal causative agent, Candida intrauterine inflammation caused by *Candida* is a rare but recognized cause of preterm birth [22]. Therefore, we hypothesized that intrauterine fungal infection was one of the reasons for the higher detection rate of fungal infection in VLBWI.

The total fungal detectable rate in the experimental group increased from the first day (64.71%) to the third day (85.19%), followed by a smoothly decreasing trend, while in the control group, in contrast, gradually increased from a lower postnatal detectable rate (33.33%) to 62.5% on day 28. Candida detection rate showed a fluctuating trend within 2 months after birth, which peaked on the third day and troughs on the 21st day. Since there are few known studies using high-throughput sequencing technology for the analysis of neonatal intestinal fungi, therefore the literature available for comparison is insufficient. However, in the study [23] by Nahid Kondori et al. on the fungal cultures of Swiss infants, it was found that the Candida detectable rate from 3 days of age maintains an increase to 18 months of age; this difference with our experimental results may be caused by the different detection method [3], resulting in bias in the final results. More studies of the same experimental method are needed to compare with this experiment.

A comparison of alpha diversity between the two groups revealed a greater abundance of fungus in the VLBWI group. We speculate that the fact that VLBWI face more exposure to the environment, healthcare professionals, and the occurrence of more invasive operations (mechanical ventilation, central venous line placement, etc.) [16, 24, 25] after birth may contribute to their having a more abundance of fungus. There were differences in alpha diversity among VLBWI groups at time point. At day 60, alpha diversity was significantly higher than ever before, suggesting a significant increase in fungal diversity in the stool at day 60. In the prospective study of parent-offspring fungal colonization by Kasper Schei et al. [26], it was found that fungal alpha diversity in infants reached a minimum in 10-day samples and thereafter start increasing steadily from birth to 2 years of age. It was also found that species diversity gradually increased with infant age and diet [25]. In addition, a study [27] found that fungi were also present on the surface of the NICU environment and in breast milk. All of the above suggest that the differences in alpha diversity at different times may be related to the environment, the age itself, and the dietary intake in which the VLBWI was born.

In VLBWI, the combined abundance of Ascomycota (50.3%) and Basidiomycota (48.8%) was 99.1%, suggesting that the majority of intestinal fungus in the samples belonged to the above two phyla. This is generally consistent with the results of previous studies [2, 25, 26, 28]. At the genus level, most of the fungi in the VLBWI group consisted of a few fungal genus, with the most dominant fungal genera being *Malassezia* (21.2%) and *Candida* (10.8%). In contrast to the second-highest abundance of *Candida* found in this study, previous studies found that *Candida* ranked first in abundance among the detected intestinal fungi [2, 23, 25, 26]. *Malassezia* and *Candida* were present at different samples at different points in time, so we consider they more likely to be intestinal colonizing fungus rather than transient population. A study [2] found that the main composition of the fungal population shifted from *Malassezia* to *Candida* could be observed at 1 to 5 months, but the abundance of *Malassezia* was consistently higher than *Candida* during the period observed (postnatal to 2 months of age) in our study. A longer follow-up may be needed to verify previous observations.

In our study, in addition to the two fungal genera mentioned above, other dominant fungal genera are *Leucosporidium* (8.45%), *Cystofilobasidium* (8.11%), *Pseudogymnoascus* (6.17%), *Mycosphaerella* (3.92%), *Alternaria* (3.74%), *Cutaneotrichosporon* (3.60%), *Cladosporium* (3.54%), and *Aspergillus* (3.37%). This is not exactly the same as the previous studies by James et al. [25]. Previous studies found that the composition and abundance of intestinal fungi varied greatly between individuals [13, 25, 29], and symbiotic fungal populations are more variable than bacterial populations [30]. Some of the fungal genera found in these studies on neonates were similar to adults [3, 31, 32]. In the Human Microbiome Project, ITS2 and 18S rRNA sequencing results indicated that *Saccharomyces*, *Malassezia*, *Candida*, *Cyberlindnera*, *Penicillium*, *Cladosporium*, *Aspergillus*, *Debaryomyces*, *Pichia*, *Clavispora*, and *Galactomyces* are the most common fungal genera in the human gut [29]. Based on the above observation, we speculate that the fungal community in the gut during the earliest stages of life has an influence on the composition of the gut fungi in adults.

The most basic condition for fungal colonization in humans is the ability to survive at a temperature of 37 °C. Some of the detected genera of fungi, such as *Cladosporium*, are not able to grow at human temperatures, and its ability to produce spores suggests that it may be caused by the inhalation of spores present in the environment by the host [25]. Many fungi are usually present in soil and air and bound to plants as pathogens or saprophytes and enter the digestive tract by ingestion (as foodborne contaminants) or inhalation [25]. In our study, some fungi such as Sonoraphlyctis, Anthracocystis, and Thecaphora occur only a few times in very low abundance, so we consider that they are of environmental origin and are not able to colonize the intestine.

The VLBWI group showed enhanced D-myo-inositol (1,4,5)-trisphosphate biosynthesis. This pathway mediates the biological response of a large number of hormones and neurotransmitters in target cells by regulating calcium release from intracellular stores and make roles in controlling calcium homeostasis, transferring calcium between intracellular stores, and regulating calcium entry across the plasma membrane [33], while the expression of all other metabolic functions was diminished. Rozlyn et al. [13] reported that at 1 year of age, fungal communities in the infant gut demonstrate an increased capacity for functions related to energy metabolism and a decrease in degradation pathways relative to communities at 3 months of age, but no more experiments were performed to verify this finding.

In conclusion, this study describes the composition and dynamics of intestinal fungi over time during the first 2 months of life in VLBWI and provides information for future studies on the intestinal fungus of children. However, there are some limitations in this study: firstly, we can not determine whether the detected fungus is intestinal colonization or temporary foreign contamination, which requires more study subjects and a longer observation period to verify. Since fungi are ubiquitous in the environment, and fungal DNA represents a relatively low percentage of fecal DNA content (especially in meconium), rigorous extraction, purification, and amplification techniques are required [2], and it is difficult to differentiate environmental fungi from colonizing fungi from the sample. A prominent reason why the study of fungi lagging behind that of the microbiome is the lack of standardization of fungal bioassay methods [29]. Secondly, we did not include full-term newborns as a separate control group, so we cannot compare VLBWI with healthy infant for further analysis. Finally, the observation period of this study was only 2 months after the birth of the study subjects which is too short to identify and summarize characteristics. In the future, we will select full-term infants as a control group and extend the observation period to explore the correlation and influence of the establishment, change, and maturation of intestinal fungal community with some clinical factors, such as gestational age, sex, birth weight, invasive manipulation, and food intake. We also tried to further analyze the relationship between intestinal fungi and disease occurrence in the context of clinical diseases.

## Data Availability

The datasets used and analyzed during the current study are available from the corresponding author on reasonable request.

## Abbreviations

ASVs: Amplicon Sequence Variants
FI: Feeding intolerance
LOS: Late-onset sepsis
NEC: Necrotizing enterocolitis
NICU: Neonatal Intensive Care Unit
VLBWI: Very low birth weight infants
